# Elevated Serum Hepcidin Levels during an Intensified Training Period in Well-Trained Female Long-Distance Runners

**DOI:** 10.3390/nu9030277

**Published:** 2017-03-14

**Authors:** Aya Ishibashi, Naho Maeda, Daichi Sumi, Kazushige Goto

**Affiliations:** 1 Department of Sports Science, Japan Institute of Sports Science, Nishigaoka, Kitaku, Tokyo 115-0056, Japan; aya.ishibashi@jpnsport.go.jp; 2 Graduate School of Sport and Health Science, Ritsumeikan University, Kusatsu, Shiga 525-8577, Japan; gr0237rr@ed.ritsumei.ac.jp (N.M.); sh0034vr@ed.ritsumei.ac.jp (D.S.)

**Keywords:** iron homeostasis, athletes, training volume

## Abstract

Iron is essential for providing oxygen to working muscles during exercise, and iron deficiency leads to decreased exercise capacity during endurance events. However, the mechanism of iron deficiency among endurance athletes remains unclear. In this study, we compared iron status between two periods involving different training regimens. Sixteen female long-distance runners participated. Over a seven-month period, fasting blood samples were collected during their regular training period (LOW; middle of February) and during an intensified training period (INT; late of August) to determine blood hematological, iron, and inflammatory parameters. Three-day food diaries were also assessed. Body weight and lean body mass did not differ significantly between LOW and INT, while body fat and body fat percentage were significantly lower in INT (*p* < 0.05). Blood hemoglobin, serum ferritin, total protein, and iron levels, total iron-binding capacity, and transferrin saturation did not differ significantly between the two periods. Serum hepcidin levels were significantly higher during INT than LOW (*p* < 0.05). Carbohydrate and iron intakes from the daily diet were significantly higher during INT than LOW (*p* < 0.05). In conclusion, an elevated hepcidin level was observed during an intensified training period in long-distance runners, despite an apparently adequate daily intake of iron.

## 1. Introduction

Because iron is essential for oxygen transport to working muscles, it plays an important role in energy production during exercise [1]. Iron deficiency leads to a decreased exercise capacity during endurance events [2,3]. Among endurance athletes, iron deficiency is prevalent as a result of their prolonged training and repetitive ground impact [4]. Several physiological mechanisms have been proposed to explain the impaired iron status, including gastrointestinal bleeding [5], hemolysis [6], lack of iron in the daily diet [7], and loss via sweat [8]. However, the detailed mechanisms underlying exercise-induced iron deficiency among athletes remain unclear.

Hepcidin, a 25-amino-acid peptide hormone, is a key mediator of iron homeostasis [9], and it may represent another mechanism of iron deficiency in response to exercise training [10,11,12]. Hepcidin regulates iron balance by binding to the iron export protein ferroportin, which inhibits iron efflux from enterocytes, hepatocytes, and macrophages [13]. Hepcidin expression is upregulated by increased iron stores [14] and inflammation [15]. Because pro-inflammatory cytokines, such as interleukin-6 (IL-6), also stimulate hepcidin production, sustained inflammation may increase hepcidin, resulting in iron deficiency and anemia [16,17].

Strenuous exercise causes inflammation, and a marked increase in IL-6 levels is observed after prolonged exercise [18], which stimulates hepcidin production. Serum hepcidin levels are elevated around 3–6 h after a single bout of exercise [11,19,20,21]. However, information about the cumulative effect of daily training on hepcidin levels in female athletes is still limited. Although an increased prevalence of iron depletion was found previously during a competitive season in endurance athletes [22], whether an increased training volume augments hepcidin levels in female athletes has not been fully examined.

Thus, the purpose of the present study was to compare serum hepcidin levels and iron metabolism between two periods involving different training volumes in female long-distance runners. We hypothesized that increased training would elevate serum hepcidin levels.

## 2. Materials and Methods

### 2.1. Subjects

In total, 16 female long-distance runners (top level of university runners in Japan) (mean ± standard deviation age: 20.5 ± 1.0 years) participated. All subjects belonged to the same track and field team at a university and lived together in a dormitory for the long-distance running team. They regularly performed training twice per day (1 h in the early morning, 2 h in the afternoon) six days per week. The subjects were informed about the purpose and procedures of the study, and written informed consent was obtained from all subjects. This study was approved by the Ethics Committee for Human Experiments at the Ritsumeikan University (BKC-IRB-2014-025), Japan.

### 2.2. Experimental Design

**Figure 1:**
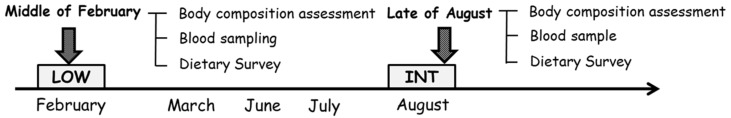
Experimental design during LOW and INT.

To evaluate the influence of different training regimens on iron metabolism, blood drawings and dietary surveys were performed twice over a period of seven months (Figure 1): during a regular training period (LOW; middle of February 2015) and an intensified training period (INT; late of August 2015). We have compared iron metabolism between these periods, because running distances during LOW and INT were apparently different. Moreover, the training menu during each period was maintained over a month, and iron metabolism evaluated during each period was considered to be relatively stable at each time point. The subjects reported average monthly running distances. On the day of measurement, recent use of iron and other supplements and menstrual cycle status were noted. In addition to body composition, hematological parameters, iron and inflammatory parameters in blood and dietary intake were assessed. Body composition measurement and blood sampling were taken before breakfast, and all subjects did not consume any fluid prior to the measurements (6:30–8:00 a.m.).

### 2.3. Measurement Procedures

#### 2.3.1. Body Composition

Body mass and fat mass were evaluated using a multi-frequency impedance technique (InBody 720, Biospace, Seoul, Korea). Using a range of frequencies from 1 kHz to 1 MHz, the InBody 720 accurately measures the amount of body water and body composition, including fat, free fat, and skeletal muscle masses [23,24]. Subjects emptied their bladders prior to the measurements.

#### 2.3.2. Blood Sampling and Analysis

Blood samples were collected following an overnight fast (6:30 p.m. to 8:00 a.m.). All blood samples were obtained via an antecubital vein while in a seated position. After drawing blood, serum and plasma samples were obtained after centrifugation (3000 rpm, 10 min, 4 °C) and stored at −80 °C until analyzed.

The hematological parameters of blood hemoglobin (Hb) levels, mean corpuscular volume (MCV), mean corpuscular hemoglobin (MCH), and mean corpuscular hemoglobin concentration (MCHC) were measured at a clinical laboratory (Falco Holdings, Kyoto, Japan). Serum iron, ferritin, and total protein levels, total iron-binding capacity (TIBC), and creatine kinase (CK) levels were also evaluated at a clinical laboratory (SRL, Tokyo, Japan). Transferrin saturation (TSAT) was calculated as the serum iron level/serum total iron binding capacity ×100 [25]. Plasma IL-6 and serum hepcidin level were determined by enzyme-linked immunosorbent assay (ELISA) using commercially available kits (R & D Systems Inc., Minneapolis, MN, USA). All samples for ELISA were analyzed in duplicate, and average values were determined. The interassay coefficients of variation were 1.4% (iron), 3.8% (ferritin), 1.3% (total protein), 2.0% (TIBC), 2.7% (CK), 2.3% (IL-6), and 3.1% (hepcidin).

#### 2.3.3. Dietary Assessment

Dietary surveys using three-day food diaries (3dFDs) along with verbal and written instructions for accurate recording of all foods and fluids consumed were conducted. The subjects were also requested to take photos and weigh their plates before and after a meal. The 3dFD included two week days (training days) and one weekend day (non-training day) to account for variability over a week. Once the subjects submitted their 3dFDs, a dietitian checked the food records and confirmed the contents, clarifying specific items and/or detailed information as necessary. Dietary analysis of the 3dFD was conducted using specially designed software (Eiyo-kun, Kenpaku-sha, Tokyo, Japan). Information about nutrient supplement use was also collected during recall visits. Nutrient intake from supplements (if any) was included in the present data.

#### 2.3.4. Statistical Analyses

All experimental data are shown as means ± standard deviation. Normal distributions for each variable were assessed using the Kolmogorov-Smirnov test. An independent *t*-test was used to compare variables between LOW and INT. The statistical analyses were performed using SPSS software (ver. 22.0; SPSS Inc., Chicago, IL, USA). *p*-values < 0.05 were considered to indicate statistical significance.

## 3. Results

Monthly running distances were significantly longer during INT (622 ± 94 km/month) than during LOW (499 ± 106 km/month, *p* = 0.029). During each period, 0% (LOW) and 44% (INT) of the subjects used iron supplements. No subject used any other supplement during these periods. Regular menstrual cycles (28 ± 2 days) were observed in 25% (LOW) and 19% (INT) of the subjects. Two subjects were under amenorrhea with irregular menstrual cycles.

### 3.1. Body Composition

Body weight and lean body mass did not differ significantly between LOW and INT. The body fat amount and body fat percentage were significantly lower in INT (*p* = 0.015 and 0.023, respectively; Table 1).

### 3.2. Iron Status Assessment

Comparisons of hematological variables between the two periods are shown in Table 2. Blood Hb, MCV, MCH, MCHC, serum ferritin, total protein, iron, TIBC, and TSAT did not differ significantly (*p* > 0.05 for all variables). Serum CK levels tended to be higher during INT (*p* = 0.09). Plasma IL-6 levels did not differ significantly between the two periods. None of subjects were regarded as iron-deficient with anemia. However, among the 16 subjects, 31% (LOW) and 37% (INT) of subjects were found to be iron-deficient (serum ferritin levels < 20 ng/mL) [26].

### 3.3. Serum Hepcidin Levels

**Figure 2:**
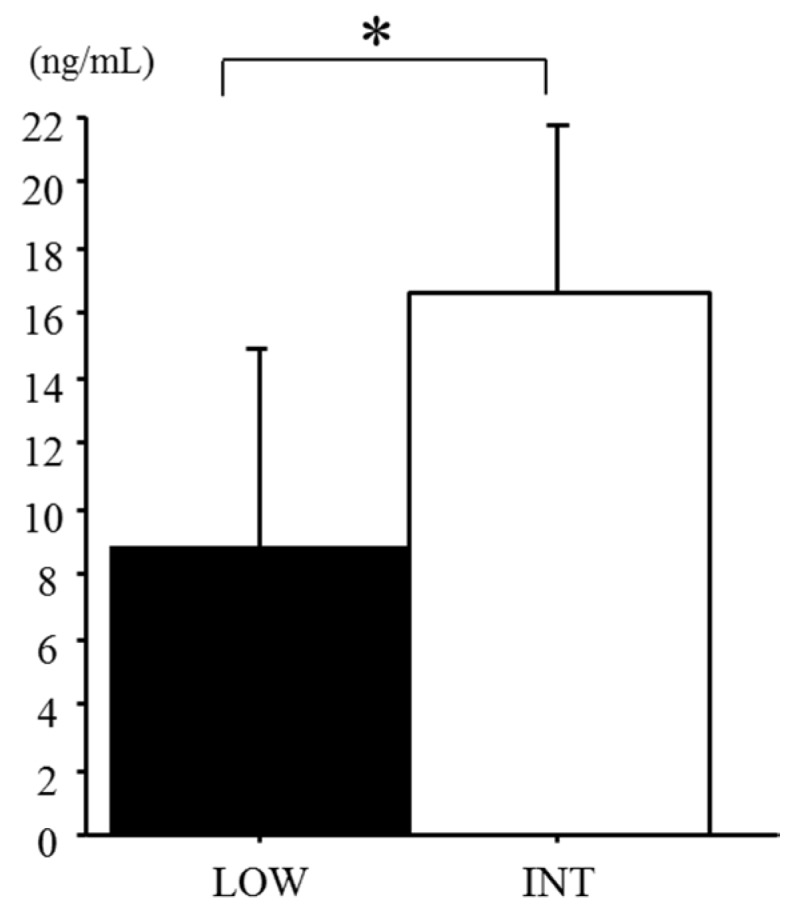
Comparison of serum hepcidin levels between LOW and INT. Values are means ± SD. * *p* < 0.05 between the periods.

**Figure 3:**
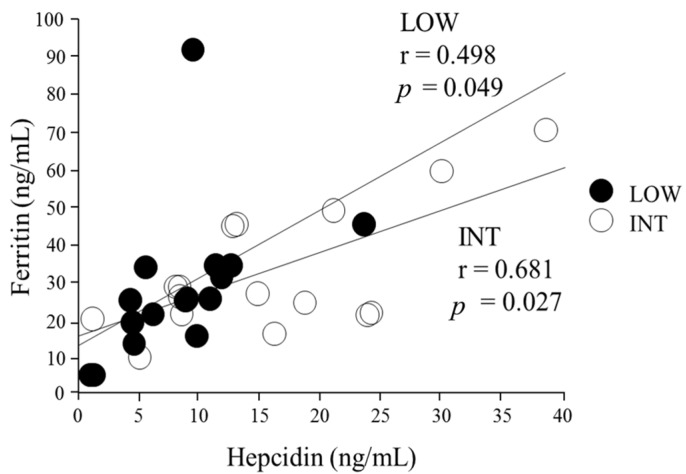
Correlation between serum hepcidin and ferritin levels.

Serum hepcidin levels were significantly higher during INT than during LOW (LOW 8.8 ± 6.1 ng/mL, INT 16.3 ± 6.5 ng/mL, *p* = 0.027; Figure 2). Several subjects revealed an irregular menstrual cycle, but it did not markedly affect serum hepcidin levels in either LOW or INT. A positive correlation was found between serum hepcidin and ferritin levels during both LOW (*r* = 0.498, *p* = 0.049) and INT (*r* = 0.681, *p* = 0.027; Figure 3). When the influence of the iron supplement on the serum hepcidin levels was determined, there was no significant difference in serum hepcidin levels between the iron-supplemented subjects and the iron–non-supplemented subjects (*p* = 0.10).

### 3.4. Energy and Macronutrient Intake

Comparisons of energy and macronutrient intakes during the three days between the two periods are shown in Table 3. Carbohydrate and iron intakes from the daily diet were significantly higher during INT (*p* = 0.002 and 0.047, respectively). Iron intake was calculated, including iron supplements. Among the subjects with iron supplements, the average amount of the iron supplements was 9.5 ± 3.0 mg (*n* = 7). The Energy intake tended to be higher during INT than LOW (*p* = 0.052). In contrast, protein and fat intakes were significantly lowered during INT (*p* = 0.002 and 0.010, respectively).

## 4. Discussion

The major finding in this study was that serum hepcidin levels were elevated significantly with an increase in the monthly running distance (INT) in female long-distance runners. 

Several physiological mechanisms have been suggested to explain exercise-induced impairment of iron status. In addition to the typically reported factors, excessive physical activity may aggravate the iron status via increased hepcidin levels [12,27]. In the present study, we observed that elevated serum hepcidin levels were associated with increased training intensity. This is consistent with results indicating that serum hepcidin levels were elevated in young tennis players during the tournament season [28]. Dzedzej et al. reported that hepcidin and IL-6 levels were significantly higher in professional basketball players than in non-athletes at the end of the season [29]. Moreover, seven days of running training increased basal urinary hepcidin levels significantly [30]. Thus, increased training seems likely to augment hepcidin secretion. In the present study, well-trained female long-distance runners (top level of university runners in Japan) were recruited, and they were regularly involved in daily training twice a day, approximately 18 h per week. Since most elite athletes perform more than one training session within a day, it is considered that the present findings can be widely applicable for athletes with iron deficiency in different types of endurance events.

In previous studies involving cell culture [15,31] and animals [32], IL-6 stimulated hepcidin biosynthesis. Pedersen and Hoffman-Goetz also demonstrated that strenuous exercise induces a marked increase in IL-6 levels [4]. The detailed mechanism of the exercise-induced hepcidin response is not fully understood, but previous studies revealed that acute exercise initially increases IL-6 and subsequently hepcidin levels 3 h after the exercise [11,19,20]. Unfortunately, we were not able to determine the influence of exercise-induced IL-6 elevation on hepcidin responses. Based on prevalent reports that demonstrated that exercise acutely increased IL-6 [18], we evaluated plasma IL-6 levels at baseline only, not after exercise. However, exercise-induced acute elevation of IL-6 during daily training may cause sustained elevation of hepcidin with a concomitant increase in the risk of iron deficiency. 

In the present study, 31% (LOW) and 37% (INT) of subjects were classified as iron-deficient (serum ferritin level < 20 ng/mL). Ferritin levels were reduced in female endurance athletes [22] and professional soccer players [33] during the competitive seasons. Somewhat surprisingly, a significant positive correlation was observed between serum hepcidin and ferritin levels during both LOW and INT. However, caution should be taken with the interpretation of this correlation, because acute inflammation increases ferritin levels [34], and increased ferritin levels due to sustained inflammation might augment hepcidin levels to maintain iron homeostasis [35]. Recently, Dzedze et al. suggested that baseline hepcidin levels were correlated significantly with serum ferritin levels in basketball players during the competitive season [29]; however, the relationship was not evident at the end of the season. Serum ferritin is commonly considered an indicator of iron status; elevated ferritin levels during an intensified training period may reflect sustained inflammation.

A strength of the present study was the dietary assessment conducted, whereas most previous studies failed to present detailed information regarding daily dietary intake. Nuviala et al. reported that the energy intake in female athletes with iron deficiency (serum ferritin level <20 ng/mL) was 2176 ± 530 kcal/day. In the present study, the daily energy intake was 2140 ± 130 kcal/day (LOW) and 2318 ± 343 kcal/day (INT) [36]. Unfortunately, we were not able to evaluate energy availability due to the lack of data on energy expenditure during training. In the present study, serum hepcidin levels tended to be higher in subjects with iron supplementation. Although the efficacy of iron supplementation on performance improvement is not fully evident, iron overload has been reported in competitive athletes [37]. A high dose of iron supplementation may stimulate hepcidin production to maintain iron homeostasis [38]. In the present subjects, the average iron intake was 14.4 ± 0.4 mg/day (LOW) and 17.3 ± 1.3 mg/day (INT), respectively. These values were above or close to previously reported values (e.g., 14 mg/day) [39,40]. Considering that a recommendation for iron intake in female endurance runners is 18 mg/day [41], the amount of iron intake in the present subjects was moderate.

Several limitations should be considered in interpreting these results. First, we did not have a control group (group not in training), because all subjects were competitive athletes. Second, energy expenditure during training was not assessed. Pasiakos et al. reported that changes in hepcidin levels after a four-day military training regimen were related to the total daily energy expenditure [42]. Thus, 24 h energy expenditure data will be of value in determining whether lower energy availability is a predominant factor in elevated hepcidin levels during the intensified training period. 

From a practical viewpoint, the present findings may suggest a cumulative effect of daily endurance training on hepcidin elevation in well-trained female endurance athletes. Since hepcidin levels were associated with increased training volume (running distance), athletes and coaches should pay attention to iron status during intensified training periods. Future studies are required to confirm whether nutrition intervention (e.g., increased carbohydrate intake, appropriate energy availability) will attenuate hepcidin elevation and the risk of iron deficiency in female endurance athletes.

## 5. Conclusions

Elevated hepcidin levels were observed during an intensified training period in female long-distance runners, despite an apparently adequate daily iron intake. Further research is needed to determine the influence of increased training intensity and/or volume on exercise-induced hepcidin responses as well as baseline levels. 

## Figures and Tables

**Table 1:** Comparisons of physical characteristics over experiment period.

Variables	LOW	INT	*p*
Height (cm)	160.5 ± 3.9	-	-
Body weight (kg)	49.7 ± 5.1	49.1 ± 4.0	N.S.
Lean body mass (kg)	41.4 ± 3.4	41.7 ± 2.0	N.S.
Body fat (kg)	8.0 ± 2.9	7.4 ± 2.3	0.015
% Body fat (%)	15.8 ± 4.9	15.0 ± 4.3	0.023

Values are means ± SD.

**Table 2:** Comparisons of blood variables over experiment period.

Variables	LOW	INT	*p*
Hb (g/dL)	12.9 ± 0.8	13.4 ± 0.1	N.S.
MCV (μm^3^)	90.0 ± 2.8	92.7 ± 3.0	N.S.
MCH (pg)	30.6 ± 2.6	30.7 ± 1.0	N.S.
MCHC (g/dL)	32.1 ± 0.7	33.1 ± 0.7	N.S.
Ferritin (ng/mL)	30.9 ± 22.2	28.1 ± 11.8	N.S.
Total Protein (g/dL)	7.2 ± 0.4	7.1 ± 0.3	N.S.
Iron (μg/dL)	55 ± 24	65 ± 8	N.S.
TIBC (μg/dL)	340.7 ± 44.6	323.0 ± 6.9	N.S.
TSAT (%)	16.4 ± 7.5	20.1 ± 2.4	N.S.
CK (IU)	227 ± 110	369 ± 66	0.09
IL-6 (pg/mL)	0.35 ± 0.23	0.33 ± 0.23	N.S.

Values are means ± SD. Abbreviations presents as follow; Hemoglobin (Hb), mean corpuscular volume (MVC), mean corpuscular hemoglobin (MCH), mean corpuscular hemoglobin concentration (MCHC), total iron-binding capacity (TIBC), transferrin saturation (TSAT), creatine kinase (CK), interleukin (IL).

**Table 3:** Comparisons of energy and macronutrient intakes during the three-day training period.

Variables		LOW	INT	*p*
Energy	(kcal)	2140 ± 130	2318 ± 343	0.052
	(KJ)	8958 ± 544	9703 ± 1437	0.052
	(kcal/BWkg)	44 ± 5	48 ± 4	0.034
Protein	(g)	115.8 ± 9.7	103.5 ± 17.9	0.002
	(g/BWkg)	2.4 ± 0.4	2.1 ± 0.5	<0.001
Fat	(g)	64.2 ± 13.5	54.2 ± 0.6	0.010
Carbohydrate	(g)	275.8 ± 31.2	353.0 ± 75.4	0.002
	(g/BWkg)	5.6 ± 1.0	7.2 ± 1.5	<0.001
Iron	(mg)	14.4 ± 1.6	17.3 ± 5.4	0.047
Vitamin C	(mg)	228 ± 53	243 ± 94	N.S.

Values are means ± SD.

## Abbreviations

IL-6	interleukin-6
Hb	hemoglobin
MCV	mean corpuscular volume
MCH	mean corpuscular hemoglobin
MCHC	mean corpuscular hemoglobin concentration
TIBC	total iron-binding capacity
CK	creatine kinase
TSAT	transferrin saturation
CV	coefficients of variation
